# Investigation of metabolic features of glioblastoma tissue and the peritumoral environment using targeted metabolomics screening by LC-MS/MS and gene network analysis

**DOI:** 10.18699/vjgb-24-96

**Published:** 2024-12

**Authors:** N.V. Basov, A.V. Adamovskaya, A.D. Rogachev, E.V. Gaisler, P.S. Demenkov, T.V. Ivanisenko, A.S. Venzel, S.V. Mishinov, V.V. Stupak, S.V. Cheresiz, O.S. Oleshko, E.A. Butikova, A.E. Osechkova, Yu.S. Sotnikova, Y.V. Patrushev, A.S. Pozdnyakov, I.N. Lavrik, V.A. Ivanisenko, A.G. Pokrovsky

**Affiliations:** N.N. Vorozhtsov Novosibirsk Institute of Organic Chemistry of the Siberian Branch of the Russian Academy of Sciences, Novosibirsk, Russia Novosibirsk State University, Novosibirsk, Russia; Novosibirsk State University, Novosibirsk, Russia Institute of Cytology and Genetics of the Siberian Branch of the Russian Academy of Sciences, Novosibirsk, Russia; N.N. Vorozhtsov Novosibirsk Institute of Organic Chemistry of the Siberian Branch of the Russian Academy of Sciences, Novosibirsk, Russia Novosibirsk State University, Novosibirsk, Russia; Novosibirsk State University, Novosibirsk, Russia; Novosibirsk State University, Novosibirsk, Russia Institute of Cytology and Genetics of the Siberian Branch of the Russian Academy of Sciences, Novosibirsk, Russia; Novosibirsk State University, Novosibirsk, Russia Institute of Cytology and Genetics of the Siberian Branch of the Russian Academy of Sciences, Novosibirsk, Russia; Novosibirsk State University, Novosibirsk, Russia Institute of Cytology and Genetics of the Siberian Branch of the Russian Academy of Sciences, Novosibirsk, Russia; Novosibirsk Research Institute of Traumatology and Orthopedics named after Ya.L. Tsivyan of the Ministry of Health of the Russian Federation, Novosibirsk, Russia; Novosibirsk Research Institute of Traumatology and Orthopedics named after Ya.L. Tsivyan of the Ministry of Health of the Russian Federation, Novosibirsk, Russia; Novosibirsk State University, Novosibirsk, Russia; Novosibirsk State University, Novosibirsk, Russia; Novosibirsk State University, Novosibirsk, Russia Research Institute of Clinical and Experimental Lymрhology – Branch of the Institute of Cytology and Genetics of the Siberian Branch of the Russian Academy of Sciences, Novosibirsk, Russia; N.N. Vorozhtsov Novosibirsk Institute of Organic Chemistry of the Siberian Branch of the Russian Academy of Sciences, Novosibirsk, Russia Boreskov Institute of Catalysis of the Siberian Branch of the Russian Academy of Sciences, Novosibirsk, Russia; N.N. Vorozhtsov Novosibirsk Institute of Organic Chemistry of the Siberian Branch of the Russian Academy of Sciences, Novosibirsk, Russia Novosibirsk State University, Novosibirsk, Russia Boreskov Institute of Catalysis of the Siberian Branch of the Russian Academy of Sciences, Novosibirsk, Russia; A.E. Favorsky Irkutsk Institute of Chemistry of the Siberian Branch of the Russian Academy of Sciences, Irkutsk, Russia Boreskov Institute of Catalysis of the Siberian Branch of the Russian Academy of Sciences, Novosibirsk, Russia; A.E. Favorsky Irkutsk Institute of Chemistry of the Siberian Branch of the Russian Academy of Sciences, Irkutsk, Russia; Institute of Cytology and Genetics of the Siberian Branch of the Russian Academy of Sciences, Novosibirsk, Russia; Novosibirsk State University, Novosibirsk, Russia Institute of Cytology and Genetics of the Siberian Branch of the Russian Academy of Sciences, Novosibirsk, Russia Kurchatov Genomic Center of ICG SB RAS, Novosibirsk, Russia; Novosibirsk State University, Novosibirsk, Russia

**Keywords:** glioblastoma, peritumoral tissue, markers, metabolomics, LC-MS/MS, sphingolipids, metabolic pathways, gene networks, cognitive system ANDSystem, глиобластома, перитуморальная ткань, маркеры, метаболомика, ВЭЖХ-МС/МС, сфинголипиды, метаболические пути, генные сети, когнитивная система ANDSystem

## Abstract

The metabolomic profiles of glioblastoma and surrounding brain tissue, comprising 17 glioblastoma samples and 15 peritumoral tissue samples, were thoroughly analyzed in this investigation. The LC-MS/MS method was used to analyze over 400 metabolites, revealing significant variations in metabolite content between tumor and peritumoral tissues. Statistical analyses, including the Mann–Whitney and Cucconi tests, identified several metabolites, particularly ceramides, that showed significant differences between glioblastoma and peritumoral tissues. Pathway analysis using the KEGG database, conducted with MetaboAnalyst 6.0, revealed a statistically significant overrepresentation of sphingolipid metabolism, suggesting a critical role of these lipid molecules in glioblastoma pathogenesis. Using computational systems biology and artificial intelligence methods implemented in a cognitive platform, ANDSystem, molecular genetic regulatory pathways were reconstructed to describe potential mechanisms underlying the dysfunction of sphingolipid metabolism enzymes. These reconstructed pathways were integrated into a regulatory gene network comprising 15 genes, 329 proteins, and 389 interactions. Notably, 119 out of the 294 proteins regulating the key enzymes of sphingolipid metabolism were associated with glioblastoma. Analysis of the overrepresentation of Gene Ontology biological processes revealed the statistical significance of 184 processes, including apoptosis, the NF-kB signaling pathway, proliferation, migration, angiogenesis, and pyroptosis, many of which play an important role in oncogenesis. The findings of this study emphasize the pivotal role of sphingolipid metabolism in glioblastoma development and open new prospects for therapeutic approaches modulating this metabolism.

## Abstract (ru)

В ходе исследования проведен комплексный анализ метаболомных профилей глиобластомы и прилегающей ткани головного мозга, включавший 17 образцов глиобластомы и 15 образцов перитуморальной ткани. С использованием метода ВЭЖХ-МС/МС было проанализировано более 400 метаболитов, что позволило выявить значимые различия в их содержании между опухолевой и перитуморальной тканями. Статистический анализ, включавший тесты Манна–Уитни и Куккони, показал, что существенное количество метаболитов, в частности церамиды, значимо различается в тканях глиобластомы и перитуморального пространства. Анализ метаболических путей из базы данных KEGG, выполненный с помощью MetaboAnalyst 6.0, выявил статистически значимую перепредставленность метаболизма сфинголипидов, что указывает на важную роль этих липидных молекул в патогенезе глиобластомы. С использованием методов компьютерной системной биологии и искусственного интеллекта, реализованных в когнитивной системе ANDSystem, реконструированы молекулярно-генетические регуляторные пути, описывающие потенциальные механизмы нарушения функции ферментов метаболизма сфинголипидов. Реконструированные пути были объединены в регуляторную генную сеть. Данная сеть включала 15 генов, 329 белков и 389 взаимодействий, при этом 119 из 294 белков, регулирующих ключевые ферменты сфинголипидного метаболизма, оказались ассоциированы с глиобластомой. Анализ перепредставленности биологических процессов Gene Ontology показал статистическую значимость 184 процессов, в том числе апоптоза, сигнального пути NF-κB, пролиферации, миграции, ангиогенеза и пироптоза, многие из которых играют важную роль в онкогенезе. Результаты исследования подчеркивают ключевую роль метаболизма сфинголипидов в развитии глиобластомы и открывают новые перспективы для разработки терапевтических подходов, направленных на его модуляцию.

## Introduction

Glioblastoma (GBM) is the most prevalent primary brain tumor in adults, with its aggressiveness primarily dictated by its invasive nature – active infiltration of individual or clustered malignant cells into the brain parenchyma surrounding the tumor (Vollmann-Zwerenz et al., 2020). The World Health Organization (WHO) classifies gliomas based on cell type and aggressiveness: grade I includes benign tumors, while grade IV encompasses the most aggressive tumor types, including glioblastomas (Louis et al., 2021). Poor survival rates among GBM patients, even after the most radical surgeries to remove the primary tumor accompanied by multimodal chemoradiotherapy (Omuro, DeAngelis, 2013), are linked to the reappearance of malignant growths. These often occur directly within the postoperative cavity, in its 2-cm marginal zone, or as distant and multiple recurrent tumor foci. Such recurrent tumors are believed to form from GBM cells in the peritumoral zone that re-migrate back into the primary tumor cavity or to distant areas of the brain.

Despite established approaches to disease verification, the challenge of predicting tumor growth and sensitivity to treatment remains unresolved. In 2016, the WHO introduced a new classification system for brain tumors, incorporating genetic markers such as IDH1/IDH2, O-6-methylguanine DNA methyltransferase (MGMT), and epidermal growth factor receptors (EGFR) (Louis et al., 2021). This system enables clinicians to differentiate tumors not only by cell type and aggressiveness, as was possible with previous methods, but also by the genetic phenotype of neoplastic cells, offering a stronger correlation with tumor prognosis (Jaroch et al., 2021). Molecular biomarkers have become an essential component of glial tumor evaluation, influencing clinical decisions in various glioma subtypes, including treatment strategies. The potential for glioma classification based on molecular markers continues to be explored, promising better implementation of personalized therapeutic approaches (Siegal, 2015). Additionally, the use of omics technologies, such as metabolomic screening, represents an exciting avenue of contemporary research aimed at identifying disease biomarkers.

Like most malignancies, glioblastoma exhibits a unique bioenergetic state of aerobic glycolysis, known as the Warburg effect (Siegal, 2015), in which aerobic glycolysis serves as the primary source of ATP for cancer cells (Warburg, 1956). Although the understanding of cancer cell metabolism is continually evolving, the specific advantages that cancer cells gain from metabolic transformation remain unclear (Koppenol et al., 2011). Additionally, the mechanisms by which hypoxia influences the metabolic reprogramming of tumor cells are not yet fully understood. Recent discoveries of the connections between oncogenes and metabolic processes have reignited interest in Warburg’s findings (Poteet et al., 2013). A growing body of evidence suggests that the adaptation of aerobic glycolysis in cancer cells may contribute to biomass accumulation, thereby promoting the proliferation of malignant cells (Heiden et al., 2009). The study of tumor cell metabolism is essential for developing models that accurately reflect the composition of the tumor microenvironment (Liberti, Locasale, 2016) and for identifying new, effective therapeutic strategies. The metabolomic approach to studying glioblastoma has gained significant attention, not only as a diagnostic tool but also as a means to investigate GBM metabolism. Insights from such studies can aid in the development of novel therapeutic interventions (Pandey et al., 2017; Zhou, Wahl, 2019). In some respects, metabolomic analysis surpasses gene expression analysis, as gene function can be influenced by epigenetic modifications and post-translational changes. In contrast, metabolites act as direct indicators of enzymatic activity and biochemical processes within the cell (Pandey et al., 2017).

The analysis of metabolic differences between various regions of glioblastoma, particularly between the central region of the tumor and the peritumoral zone, is considered one of the most reliable methods for studying the tumor’s metabolic characteristics (Wolf et al., 2010; Chinnaiyan et al., 2012). These metabolic differences can be assessed using samples obtained intraoperatively during tumor resection (Youngblood et al., 2021). However, only few such studies have been reported in the literature

Various methods based on the analysis of metabolic pathways and gene networks are employed to identify molecular genetic mechanisms underlying the observed metabolomic data. Gene networks provide valuable insights into the genetic regulation of the identified metabolic pathways, forming a foundation for integrating metabolomic and genomic data (Kolchanov et al., 2013). We have previously developed ANDSystem, a software and information system designed for automated extraction of biological and medical knowledge from scientific publications using artificial intelligence methods (Demenkov et al., 2011; Ivanisenko V.A. et al., 2015, 2019; Ivanisenko T.V. et al., 2020, 2022). This software enables users to reconstruct, expand, and graphically visualize gene networks, apply data filtering, and search for regulatory pathways in the global gene network using templates. ANDSystem has been utilized to analyze molecular genetic mechanisms across a wide range of diseases, including comorbid conditions, organismal responses to stress, identification of pharmacological targets, and other research objectives (Bragina et al., 2014, 2016, 2023; Popik et al., 2016; Saik et al., 2016, 2018a, b, 2019; Zolotareva et al., 2019; Antropova et al., 2022; Demenkov et al., 2023).

Metabolomic and proteomic data have been analyzed using ANDSystem (Pastushkova et al., 2013, 2019; Binder et al., 2014; Larina et al., 2015; Rogachev et al., 2021; Ivanisenko V.A. et al., 2022, 2023). For instance, metabolomic analysis of blood plasma from COVID-19 patients identified the role of non-structural viral proteins in metabolic disorders associated with the disease, which contributed to changes in the metabolomic profile (Ivanisenko V.A. et al., 2022). Additionally, analysis of metabolomic profiles from patients with postoperative delirium, conducted using ANDSystem, helped identify potential markers represented by several sphingolipids and revealed molecular genetic mechanisms underlying their metabolic disruptions (Ivanisenko V.A. et al., 2023).

In this study, a targeted screening of a broad spectrum of metabolites was conducted in glioblastoma and peritumoral tissue. Statistical analysis of the screening data identified metabolites involved in sphingolipid metabolism, with significantly different levels observed between tumor and peritumoral tissue. Using gene network reconstruction, genes with the greatest regulatory influence on the function and expression of key enzymes in sphingolipid metabolism were identified. These included both established tumor markers (p53, TNF-α, VEGF, etc.) and promising candidate markers (KLF4, E2F4, etc.). Disruption of these genes’ functions in glioblastoma may explain the observed alterations in the metabolomic profile

## Materials and methods

**Reagents and materials.** Methanol and acetonitrile used for sample preparation and analysis were of gradient HPLC grade and were purchased from Khimmed (Moscow, Russia). Purified water was prepared using a Sartorius arium 611DI system (Göttingen, Germany). Eluent A was prepared according to the protocol described by Li et al. (2017).

**Patients – study participants.**Tumor tissue was obtained from patients who underwent surgical treatment in the neurosurgical department of the Ya.L. Tsivyan Novosibirsk Research Institute of Traumatology and Orthopedics (Novosibirsk, Russia) for first-diagnosed GBM between 2019 and 2022. The cohort included patients with grade IV gliomas hospitalized for surgical tumor resection. Diagnoses were confirmed by MRI and histopathological examination of excisional biopsy specimens. The final diagnosis was established based on histological analysis and the consensus of two pathomorphologists, following the WHO classification. Tumor samples were collected intraoperatively and anonymized for the investigators. The clinical study identifier was NCT03865355. A total of 17 patients were included in the study. Below is their gender and age distribution:

**distribution. 1.**
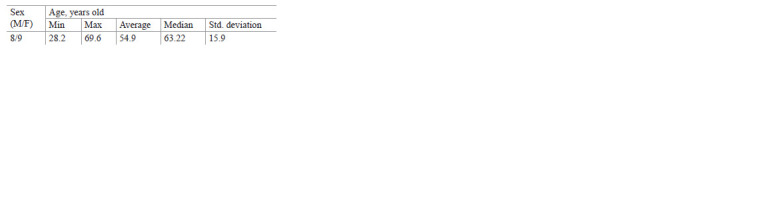
distribution1

**Collection of tumor tissue samples from patients.** Tumor tissue samples were obtained during cytoreductive surgical interventions. After collection, the samples were immediately placed in RPMI 1640 cell culture medium without additives and stored at +4 °C until processing. Tumor sections from different regions (tumor center and peritumoral tissue) were separated using surgical instruments into fragments ranging in size from 2×2×2 mm to 5×5×5 mm. These fragments were wrapped in sterile foil bags, frozen in liquid nitrogen, and subsequently stored in a low-temperature freezer at –80 °C. In total, glioblastoma samples from 17 patients and peritumoral tissue samples from 15 patients were included in the study.

**Compliance with ethical standards.**The study was reviewed and approved by the Ethics Committee of the Zelman Institute of Medicine and Psychology at Novosibirsk State University (meeting minutes dated January 4, 2018). All experimental protocols were approved, and all procedures involving human participants adhered to the ethical standards of the institutional research committee, the 1964 Declaration of Helsinki, and its subsequent amendments or equivalent ethical standards. Written informed consent was obtained from each participant prior to inclusion in the study. Additionally, the study was approved by the Local Ethical Committee of the Ya.L. Tsivyan Novosibirsk Research Institute of Traumatology and Orthopedics (meeting minutes dated September 11, 2017, No. 050/17), and informed voluntary consent was obtained from all participants.

**Sample preparation of glioblastoma and peritumoral tissue samples.**Metabolite extraction from glioblastoma and peritumoral tissue samples was carried out simultaneously using a modified protocol based on (Yuan et al., 2012; Li et al., 2017). In a 1.5 mL tube, 250 μL of chilled 80 % methanol (vol/vol) was added per 10 mg of tissue to a sample weighing between 9 and 33 mg. The samples were homogenized for 2 minutes using a Bertin Minilys tissue homogenizer (Rockville, Maryland, USA), with granite chips added to enhance sample disintegration. This was followed by incubation at –70 °C for 24 hours. After incubation, the samples were vortexed and centrifuged at +4 °C and 16,000 g for 15 minutes. The resulting supernatant was carefully transferred to a new polypropylene tube. An equal volume of chilled 80 % methanol (vol/vol) was then added to the remaining precipitate, vortexed for 1 minute, incubated at –70 °C for 30 minutes, and centrifuged under the same conditions. The supernatants from both extractions were combined, and a 500 μL aliquot was taken and evaporated to dryness using a SpeedVac concentrator vacuum centrifuge (Thermo Fisher Scientific/Savant, Waltham, USA). The dried samples were reconstituted in 20 μL of MilliQ water and subjected to analysis.

**High-performance liquid chromatography with mass spectrometric detection.**Samples were analyzed by high-performance liquid chromatography with tandem mass spectrometric detection (LC-MS/MS), following the procedure described by Basov, Rogachev et al. (2024). Chromatographic separation was performed using an LC-20AD Prominence chromatograph (Shimadzu, Japan) equipped with a SIL 20AC autosampler (Shimadzu, Japan) maintained at 10 °C. The injection volume was 2 μL. Eluent A consisted of 5 % acetonitrile in 20 mM ammonium carbonate (NH4)2CO3 aqueous solution, adjusted to pH 9.8 with aqueous ammonia solution, and eluent B was 100 % acetonitrile. Each sample was analyzed twice, in hydrophilic interaction liquid chromatography (HILIC) and reversed-phase chromatography (RP LC) modes. The HILIC gradient was as follows: 0 min – 98 % B, 2 min – 98 % B, 6 min – 0 % B, 10 min – 0 % B, followed by column equilibration for 4 min. The RP LC gradient was 0 min – 0 % B, 1 min – 0 % B, 6 min – 98 % B, 16 min – 98 % B, with column equilibration for 3 min. The flow rate for both analyses was 300 μL/min. Chromatographic analyses were performed using a monolithic column based on 1-vinyl-1,2,4-triazole (2 × 60 mm), synthesized as described by Patrushev et al. (2018) through copolymerization of styrene, divinylbenzene, and 1-vinyl- 1,2,4-triazole in a volume ratio of 10:50:40, respectively, within a glass tube with an inner diameter of 2 mm.

**Mass spectrometric detection.**Detection of 489 metabolites was performed in multiple reaction monitoring (MRM) mode as positive and negative ions using an API 6500 QTRAP mass spectrometer (AB SCIEX, USA) equipped with an electrospray ionization source operating in the positive/ negative switch mode. The primary mass spectrometric parameters were as follows: ion spray voltage (IS) was set at 5500 V for positive ionization mode and –4500 V for negative ionization mode; the ion source temperature was at 475 °C; CAD gas was set as “Medium”; GS1, GS2 and curtain gas were 33, 33 and 30 psi, respectively. The declustering potential (DP) was ±91 V, the entrance potential (EP) was ±10 V, and the collision cell exit potential (CXP) was ±9 V. In addition, the polarity switching (settling) time was set at 5 ms, and dwell time was 3 ms for each MRM transition. Precursor and fragment ion transitions, metabolite names, dwell times, and the appropriate collision energies for both positive and negative ion modes were adapted from the studies: Yuan et al. (2012) and Li et al. (2017) (Supplementary Material 1)1. Device control and data acquisition were collected using Analyst 1.6.3 software (AB SCIEX), while chromatograms were processed using Skyline 24.0 software (Adams et al., 2020).


Supplementary Materials are available in the online version of the paper: https://vavilovj-icg.ru/download/pict-2024-28/appx31.xlsx


**Pre-processing and statistical analysis of the data.** Statistical analysis of the metabolomic screening results for glioblastoma and peritumoral tissue was conducted using the Mann–Whitney and Cucconi tests, implemented in Python packages (SciPy and Nonparstat). The Mann– Whitney test was employed to identify significant differences between groups, while the Cucconi test provided additional validation of the identified differences under conditions of sample heterogeneity. Outlier correction was performed as follows: an outlier was defined as any value outside the 1.5 interquartile range (IQR). Identified outliers were replaced with adjusted values calculated as 1.5 × IQR ± 10–5 (subtracted for upper outliers and added for lower outliers).

The online platform MetaboAnalyst 6.0 (http://www. metaboanalyst.ca/) (Pang et al., 2021) and its Enrichment Analysis tool were used to identify overrepresented metabolic pathways based on highly significant metabolites.

**Reconstruction of gene networks. **Gene network reconstruction was performed using the ANDVisio graphical user interface within the ANDSystem software and information system (http://www-bionet.sscc.ru/andvisio/). In the Pathway Wizard module of ANDVisio, templates of regulatory pathways for enzymes involved in the identified metabolic pathways were created using human protein data (Table 1). The list of human protein identifiers was obtained from the SwissProt database (https://www. uniprot.org/).

**Table 1.**
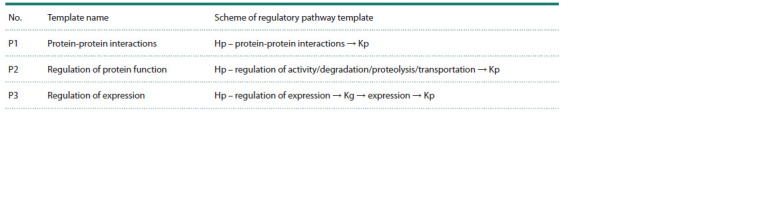
Templates of molecular genetic pathways regulating enzymes in metabolic pathways by human proteins Note. Hp – human proteins; Kg – genes encoding enzymes of the KEGG metabolic pathway; Kp – enzymes of the KEGG metabolic pathway.

The list of proteins associated with glioblastoma was retrieved from the ANDSystem knowledge base. Overrepresentation analysis of Gene Ontology biological processes was performed using the DAVID web service (https://david. ncifcrf.gov/) with default settings.

## Results

Samples of glioblastoma and brain tissue adjacent to the tumor (17 glioblastoma and 15 peritumoral tissue samples) were collected and analyzed as part of the study. Metabolomic analysis was performed using the LC-MS/MS approach developed previously (Basov et al., 2024). The chromatograms were processed by integrating the peak area of each metabolite, and the resulting signals were compared between glioblastoma and peritumoral tissue samples. Peak area values for 446 metabolites were obtained from the analysis

The Mann–Whitney test, with a critical value of p < 0.05, was used as the primary method for statistical analysis of the metabolomic screening results. The nonparametric Cucconi test was employed as an additional method for group comparisons. Metabolite lists satisfying each test at p < 0.05 were compared, resulting in a subset of metabolites that met the criteria for both tests (Table 2).

**Table 2.**
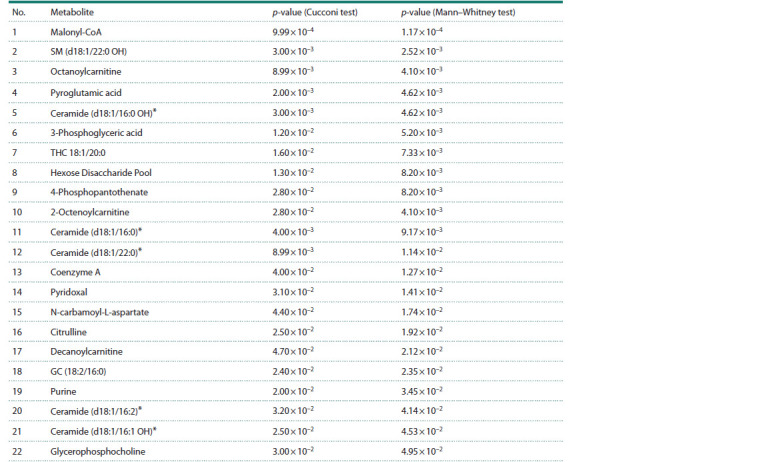
Metabolites with significant differences (p < 0.05) between glioblastoma and peritumoral samples * Metabolites belonging to the ceramide class.

The overrepresentation of KEGG metabolic pathways was analyzed for the identified set of metabolites using Metabo- Analyst 6.0 (Table 3). This analysis revealed sphingolipid metabolism as a statistically significant overrepresented metabolic pathway. Another marker-enriched pathway, the KEGG metabolic pathway “Pantothenic acid and CoA biosynthesis”, had a p-value of 0.012; however, after correction for multiple comparisons, the p-value exceeded the significance threshold of 0.05, resulting in a corrected p-value of 0.46.

**Table 3.**
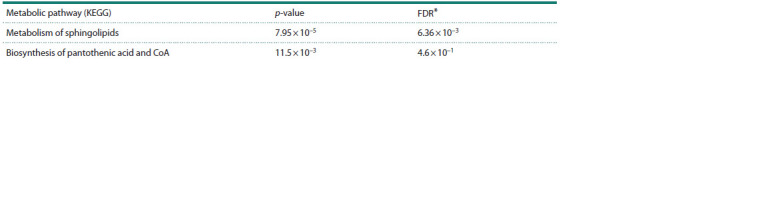
Overrepresented KEGG metabolic pathways for a set of metabolomic markers * FDR (False Discovery Rate) represents correction for multiple comparisons.

**Fig. 1.**
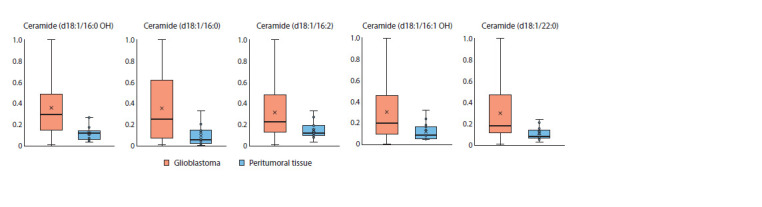
Levels of marker ceramides in tumor and peritumoral tissues

Among the metabolites identified as potential markers (Table 2), 5 out of 22 (~23 %) belonged to ceramides, a class of lipid molecules that are key components of cell membranes. Additionally, 3 metabolites – 4-phosphopantothenic acid, malonyl-CoA, and coenzyme A – were identified as major precursors in de novo lipid biosynthesis. The ceramide content was at least twofold higher in tumor tissue compared to peritumoral tissue. Furthermore, the variance was significantly greater in the glioblastoma samples, indicating higher heterogeneity within this group (Fig. 1).

**Fig. 2.**
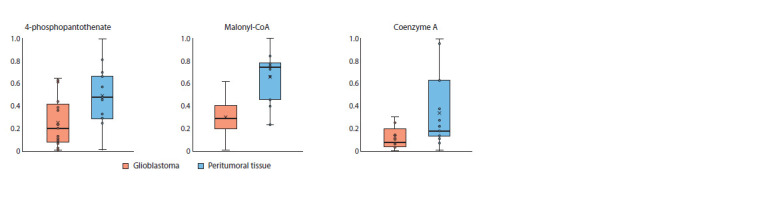
Levels of CoA and related metabolites in tumor and peritumoral tissues.

The levels of metabolites in the pantothenic acid and CoA synthesis pathway were significantly lower in tumor tissues (Fig. 2), suggesting their active utilization in lipid biosynthesis.

In the next phase of our study, we investigated potential mechanisms underlying the dysfunction of sphingolipid metabolism enzymes. To achieve this, molecular genetic regulatory pathways were reconstructed using ANDSystem with the templates presented in Table 1. These templates represent the potential regulation of enzymes involved in sphingolipid metabolism by human proteins (Supplementary Materials 2–4). The starting point of the reconstructed regulatory pathways included all human proteins, while the endpoint comprised sphingolipid metabolism enzymes from the KEGG database involved in the metabolism of ceramide, sphingomyelin (SM), glucosylceramide (GC), and trihexosylceramide (THC). For the purposes of this study, these enzymes are referred to as key enzymes of sphingolipid metabolism. The regulatory pathways considered included interactions such as protein-protein interactions, regulation of gene expression, and regulation of protein activity, degradation, or transport.

**Fig. 3.**
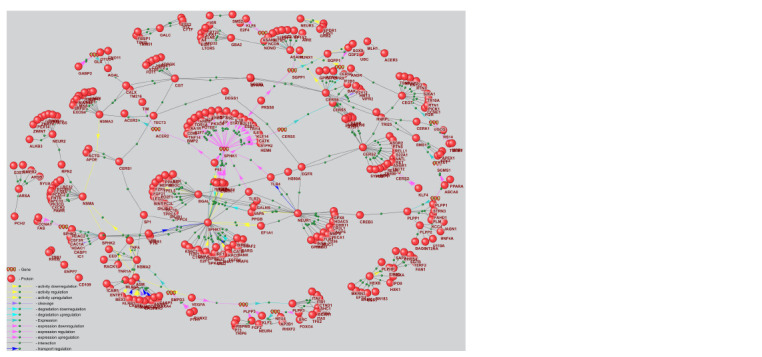
Gene regulatory network of key enzymes in sphingolipid metabolism, reconstructed by integrating regulatory pathways based on three types of templates.

The reconstructed regulatory pathways were integrated into a unified gene network (Fig. 3). This regulatory gene network comprised 15 genes, 329 proteins (including 35 enzymes involved in sphingolipid metabolism), and 389 interactions among them.

**Fig. 4.**
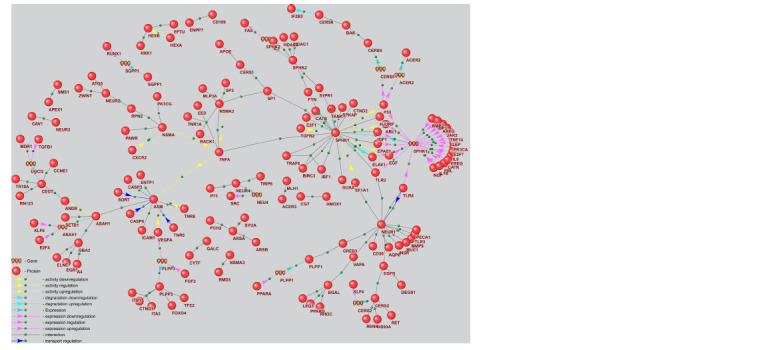
Subnetwork of the gene regulatory network for key enzymes of sphingolipid metabolism regulated by glioblastoma genetic markers

According to the ANDSystem knowledge base, evidence from the literature indicates dysfunction in glioblastoma for 119 out of 294 gene network proteins regulating key enzymes of sphingolipid metabolism. A subnetwork of the regulatory gene network, illustrating the interactions of these proteins with key enzymes of sphingolipid metabolism, is presented in Figure 4.

In total, the ANDSystem knowledge base contains association information for 2,393 human glioblastoma-related proteins, 119 of which were included in the regulatory gene network. Based on a hypergeometric test, the reconstructed gene network is statistically significantly associated with glioblastoma (p-value < 10–35).

Gene Ontology overrepresentation analysis of biological processes for genes in the resulting gene network identified 184 statistically significant processes. These include apoptosis, the NF-κB signaling pathway, proliferation, migration, and angiogenesis, which are commonly dysregulated in many cancers, as well as pyroptosis – a process, the role of which in glioblastoma is currently under active investigation (Supplementary Material 5).

## Discussion

Susceptibility of glioblastoma cells to changes in coenzyme A metabolite levels

Our study identified reduced levels of CoA and malonyl- CoA in glioblastoma tissues compared to peritumoral tissues (Fig. 2). For de novo fatty acid synthesis, glioblastoma cells must produce cytosolic acetyl-CoA, which can be generated either from citrate via ATP-citrate lyase or from acetate via acetyl-CoA synthetase (Santos, Schulze, 2012). Mashimo et al. (2014) demonstrated that brain tumors of various cellular origins have the ability to oxidize injected acetate. The authors suggest that acetate oxidation is facilitated by the activation of acetyl-CoA synthetase isoform ACSS2, achieved through upregulated expression. The higher expression of ACSS2 in glioblastoma compared to lower-grade gliomas supports the hypothesis that enzyme activation is associated with increased acetate oxidation by the tumor. Furthermore, ACSS2 deficiency in mouse models of hepatocellular carcinoma has been shown to reduce tumor burden and inhibit tumor growth (Comerford et al., 2014).

Malonyl-CoA level determines the direction of fatty acid metabolism, specifically whether it supports triglyceride synthesis or oxidation (Clarke S.D., Nakamura, 2004). Previous studies reported that inhibition of β-oxidation in human glioblastoma cells by etomoxir, a carnitine palmitoyltransferase- 1 inhibitor, significantly reduces ATP, NADPH, and reduced glutathione levels, thereby impairing cell viability (Pike et al., 2011). These findings suggest that β-oxidation contributes to oxidative stress resistance in glioblastoma cells, and our results support this hypothesis. Additionally, malonyl-CoA level has been shown to influence the response to various chemotherapeutic agents. For instance, in a study on a breast cancer cell model, malonyl-CoA levels significantly increased following fatty acid synthase inhibition and decreased upon inhibition of acetyl-CoA carboxylase (Pizer et al., 2000). Key metabolic pathways, such as glycolysis and the tricarboxylic acid (TCA) cycle, are regulated by multiple microRNAs that control specific steps within these pathways. Cancer cells predominantly rely on aerobic glycolysis instead of the TCA cycle, enabling them to sustain high ATP levels to meet biosynthetic demands (Chan et al., 2015).

Our study revealed an increase in the level of 4-phosphopantothenate, consistent with the observed changes in the lipid profile. The synthesis of this metabolite is catalyzed by pantothenate kinase, the first enzyme in the CoA biosynthetic pathway. The role of pantothenate kinase in glioblastoma has been extensively discussed in the literature. For instance, Poli et al. (2010) reported that silencing pantothenate kinase-2 significantly reduced the growth of the U373 glioma cell line. Acetyl-CoA and lipid levels may also be regulated by the microRNAs miR-103 and miR-107 (Wilfred et al., 2007). Additionally, evidence suggests that miR-103 suppresses glioblastoma cell proliferation and migration (Chen L.P. et al., 2018), while miR-107 inhibits glioblastoma angiogenesis by upregulating its expression (Chen L. et al., 2016).

Linking ceramide biosynthesis to tumor growth

Ceramides, lipid mediators of the sphingolipid class, play a role in signaling pathways that regulate cell proliferation, differentiation, and cell death (Riboni et al., 2002). Our study demonstrates that the levels of ceramides (16:0), (16:0 OH), (16:2), (16:1 OH), and (22:0) – derivatives of sphingomyelin (18:1) – are elevated in tumor tissue compared to peritumoral tissue (Fig. 1). Peritumoral tissue was used as a control because its collection during surgery does not compromise the treatment prognosis for patients. The observed increase in ceramide levels in tumor tissue suggests alterations in the enzymatic systems responsible for ceramide biosynthesis and degradation, potentially contributing to tumor growth and the evasion of apoptosis by tumor cells.

Ceramide formation occurs via three main pathways. The sphingomyelinase pathway involves the action of sphingomyelinase, an enzyme that cleaves sphingomyelin in the cell membrane to release ceramides. In the de novo synthesis pathway, ceramides are produced from simpler precursor molecules through a series of enzymatic reactions. The salvage pathway reutilizes sphingolipids by cleaving them into sphingosine, which is subsequently realkylated to form ceramide.

The key enzyme in the sphingomyelinase pathway is sphingomyelinase (SMase), which catalyzes the hydrolysis of sphingomyelin. As sphingomyelin is one of the most abundant phospholipids in the cell membrane, this pathway’s significance lies in its role in targeting the cell membrane for extracellular signals that trigger programmed cell death and cellular stress (Haimovitz-Friedman et al., 1994). SMase exists in three main types: acidic (aSMase), neutral (nSMase), and alkaline (alk-SMase). Stimulation of SMase activity can be induced by various factors, including antitumor drugs. Sphingomyelinase inhibitors, such as perphenazine and fluphenazine – classified as functional inhibitors of acidic sphingomyelinase (FIASMA) – show potential in cancer therapy, though further studies are needed to validate their efficacy (Kornhuber et al., 2010). Recent research has identified an inhibitor, Arc39, that blocks lysosomal and secretory aSMase in vitro in L929, HepG2, and B16 cells (Naser et al., 2020), as well as a light-inducible PCAI inhibitor capable of inhibiting aSMase (Prause et al., 2020). Additionally, sphingomyelinase plays a critical role in sphingolipid metabolism, which may influence cancer development (Clarke C.J. et al., 2011). Its inhibition and subsequent effects on exosomes are of growing interest for oncology and the development of therapeutic strategies (Lin M. et al., 2018).

The sphingomyelin synthase (SMS) family, comprising three members – SMS1, SMS2, and SMS-related protein (SMSr) (Chen Y., Cao, 2017) – catalyzes the synthesis of sphingomyelins from ceramides (Cer) and phosphatidylcholine, releasing diacylglycerol as a byproduct. Selective inhibition of SMS has been shown to increase ceramide concentration in the endoplasmic reticulum, triggering autophagy in hippocampal neurons (Gulbins et al., 2018). In glioblastoma, treatment with 2-hydroxyoleic acid, an antitumor drug, was observed to enhance SMS activity. Activation of SMS2 decreases ceramide levels and promotes cell proliferation via the transforming growth factor-β (TGF-β)/ Smad signaling pathway. Conversely, inhibition of SMS2 by specific miRNAs led to ceramide accumulation and accelerated cell death (Zheng et al., 2019). Recent research has shown that SMS2 is activated in breast cancer, inducing macrophage polarization and promoting tumor progression (Deng et al., 2021). Notably, SMS2 knockdown reduced the release of cytokines that drive macrophage polarization into M2 macrophages, thereby suppressing tumor growth (Deng et al., 2021). Furthermore, downregulation of SMS1 has been reported in patients with metastatic melanoma, where it is associated with worse prognosis due to an imbalance between sphingomyelin and glucosylceramide levels (Bilal et al., 2019).

Serine palmitoyltransferase (SPT) is a three subunits heteromeric enzyme that catalyzes the first step of de novo ceramide synthesis by condensing L-serine and palmitoylcoenzyme A to form 3-ketosfinganine. Increased SPT activity has been observed in response to chemotherapy and radiotherapy across various cancers. Several SPT inhibitors that block tumor growth have been identified. For instance, myriocin (ISP-1), a potent SPT inhibitor (Glaros et al., 2007), has been shown to suppress the growth of breast cancer cells (Ogretmen, 2018) and B16F10 melanoma cells by arresting the G2/M phase (Lee et al., 2011). Similar effects have been observed in human lung adenocarcinoma (HCC4006) cells, where SPT inhibition correlates with growth suppression (Sano et al., 2017). Furthermore, SPT inhibition by myriocin or specific miRNAs reduced U87MG glioblastoma cell proliferation by suppressing intracellular S1P levels (Bernhart et al., 2015). This antitumor activity is believed to result from increased levels of pro-apoptotic ceramides. In some cases, SPT activation contributes to therapeutic efficacy; for example, fenretinide, a synthetic retinoid, elevates desaturated ceramide levels, inducing apoptosis in neuroblastoma cells (Maurer et al., 1999).

Ceramide synthase (CerS) plays a role in both de novo ceramide synthesis and the salvage pathway. The CerS family comprises six isoforms, each synthesizing ceramides with specific fatty acyl-CoA chain lengths, which determine their biological activity. For instance, CerS1 produces ceramides (18:0), which inhibit tumor growth (Wang Z. et al., 2017), while CerS5 and CerS6 generate ceramides (16:0), which are associated with anti-apoptotic effects in head and neck squamous cell carcinoma (Moro et al., 2019). The CerS1-specific inhibitor P053 reduces ceramide (18:0) levels in HEK 293 cells (Turner et al., 2018). Fingolimodderived analogues (FTY720) selectively inhibit specific CerS isoforms, with inhibitors such as ST1058 and ST1074 targeting CerS2 and CerS4, while ST1072 blocks CerS4 and CerS6 activity, and ST1060 inhibits CerS2 (Schiffmann et al., 2012).

Dihydroceramide desaturase (Des1, DEGS1) is the final enzyme in de novo ceramide synthesis, converting dihydroceramide into ceramide by introducing a trans double bond at the C4-C5 position. Knockdown of Des1 by miRNAs results in cell cycle arrest in neuroblastoma cells (Kraveka et al., 2007). Resveratrol, a polyphenol with antioxidant properties, inhibits Des1 and induces autophagy in HGC27 gastric cancer cells (Signorelli et al., 2009). Other Des1 inhibitors, such as γ-tocotrienol, phenoxodiol, and celecoxib, promote autophagy by causing dihydroceramide accumulation in glioblastoma cell lines (T98G and U87MG) through Des1 inhibition (Signorelli et al., 2009). A specific Des1 inhibitor, N-[(1R,2S)-2-hydroxy-1-hydroxymethyl- 2-(2-tridecyl-1-cyclopropenyl)ethyl]octanamide, effectively activates autophagy and apoptosis in U87MG glioblastoma cells. Additionally, treatment with tetrahydrocannabinol alters the lipid composition of the endoplasmic reticulum, leading to dihydroceramide accumulation and stimulating autophagy and apoptosis in U87MG cells through reduced Des1 expression (Hernández-Tiedra et al., 2016).

Glucosylceramide synthase (GCS) is a lysosomal enzyme that glycosylates ceramides to form glycosylceramides. Elevated GCS levels have been observed in various cancers and are associated with resistance to antitumor therapies (Madigan et al., 2020).

Ceramidase is an enzyme that hydrolyzes ceramides, removing fatty acid residues to produce sphingosines. Overexpression of acidic ceramidase (ASAH1) has been detected in melanomas and is likely linked to chemotherapy resistance. ASAH1 has also been implicated in mitochondrial function and cellular autophagy in melanoma cells (Lai M. et al., 2021). Alk-SMase has been reported to play a significant role in tumor cell growth, migration, and invasion (Zhang et al., 2020). Structural analogues of ceramides have shown efficacy as selective ceramide synthase inhibitors, inhibiting cell growth and emerging as promising candidates for antitumor treatments (Steiner et al., 2016).

Disruption of genetic regulation of sphingolipid metabolism in glioblastoma

The application of ANDSystem enabled the reconstruction of a gene network describing the regulation of key enzymes involved in sphingolipid metabolism (Fig. 3). Analysis of this regulatory network revealed that 119 of its proteins are associated with glioblastoma, confirming the significant connection between the reconstructed network and this disease (p-value < 10–35). Among the most extensively studied glioblastoma-associated proteins included in the network are p53, TNF-α, TGF-β, VEGF, KLF4, and E2F4.

It is well-established that p53 is involved in numerous intracellular processes, and its dysfunction is commonly observed in various cancers. Notably, p53 plays a critical role in sphingolipid metabolism, regulating the activity of five key enzymes (CerS5, CerS6, SMPD3, ACER2, SPHK1) out of the 35 enzymes represented in the reconstructed gene network. According to Lacroix et al. (2020), p53 in tumor cells increases the expression of ceramide synthases 5 (CerS5) and 6 (CerS6) and neutral sphingomyelinase 2 (SMPD3), which are ceramide-synthesizing enzymes. Additionally, the induction of alk-SMase-2 transcription by p53 was investigated in studies by Wang Y. et al. (2017) and Xu et al. (2018).

According to the regulatory gene network, tumor necrosis factor-alpha (TNF-α) stimulates the activity of three enzymes involved in sphingolipid metabolism: acidic sphingomyelinase (ASM), neutral sphingomyelinase (NSMA), and neutral sphingomyelinase 2 (NSMA2). By enhancing the activity of these enzymes, TNF-α may facilitate sphingomyelin hydrolysis and promote ceramide formation.

Transforming growth factor-beta (TGF-β) plays a crucial role in various cell types. It initiates cellular signaling cascades that activate downstream substrates and regulatory proteins, ultimately inducing the transcription of multiple target genes. Within the regulatory gene network, TGF-β2 has been shown to enhance the activity of sphingosine kinase- 1 (SPHK1), a finding supported by Ren et al. (2009).

Vascular endothelial growth factor (VEGF) has been identified as a component of the tumor microenvironment with the capacity to activate endothelial cells. VEGF signaling operates through tyrosine kinase receptors VEGFR1 and VEGFR2, promoting endothelial cell migration, survival, proliferation, and differentiation. This process initiates angiogenesis, tumor growth, and metastasis. Within the regulatory gene network, VEGF is involved in suppressing acidic sphingomyelinase (ASM) activity and regulating the expression of phospholipid phosphatase 3 (PLPP3). Glioblastoma is characterized by a high degree of vascularization and VEGF overexpression, making this gene a compelling target for glioblastoma therapy (Tea et al., 2020).

Kruppel-like factor 4 (KLF4) is involved in regulating proliferation, differentiation, apoptosis, and somatic cell reprogramming. Evidence also indicates that KLF4 functions as a tumor suppressor in certain cancers (El-Karim et al., 2013). Within the regulatory gene network, KLF4 modulates the expression of the ceramide synthase 2 (CerS2) gene Chromatin immunoprecipitation analysis demonstrated that KLF4 directly binds to the promoter region of CerS2, activating its expression (Fan et al., 2015).

According to the reconstructed connections in the gene network, the E2F4 protein regulates the expression of ASAH1. Literature evidence indicates that E2F4 functions as a transcriptional repressor, playing a crucial role in suppressing genes associated with proliferation. Mutations and overexpression of the E2F4 gene have been linked to human cancers. By binding to the promoter region of the ASAH1 gene, E2F4 suppresses its expression (Melland-Smith et al., 2015).

Significant biological processes associated with the gene network

The overrepresented biological processes involving participants of the regulatory gene network (Supplementary Material 5) can be grouped into several categories, including programmed cell death, cell mobility, angiogenesis, and proliferation – all of which are well-documented in the context of cancer (Hanahan, Weinberg, 2000). Among these, programmed cell death via pyroptosis has garnered particular interest in recent years due to its potential role in the development and progression of glioblastoma (Lin J. et al., 2022). In the gene network, pyroptosis is represented by several caspases (CASP1, CASP3, and CASP8) and neutrophil elastase, which, under specific conditions, cleaves Gasdermin D (GSDMD) to activate pyroptosis or cleaves GSDMB, thereby inhibiting pyroptosis (Kambara et al., 2018; Oltra et al., 2023). These pyroptosis-related proteins in the gene network play a significant role in regulating this process (Rao et al., 2022) (Supplementary Material 5).

Angiogenesis is essential for providing nutrients and oxygen to glioblastoma, supporting tumor growth (Lara- Velazquez et al., 2017). Key members of the gene network, including vascular endothelial growth factor A (VEGFA), epidermal growth factor (EGF), and the catalytic subunit A of phosphatidylinositol-4,5-bisphosphate-3-kinase (PIK3CA), have been identified as important genetic markers of glioblastoma involved in angiogenesis. These genes are significant drivers of the angiogenic process (Danielsen, Rofstad, 1998). Glioblastoma is also characterized by a high capacity for invasion, with tumor cells infiltrating surrounding brain tissue, making complete surgical removal challenging and often impossible (Vollmann-Zwerenz et al., 2020). The regulatory gene network highlights proteins such as thyroid receptor-interacting protein 6 (TRIP6), which is overexpressed in glioblastoma and promotes tumor cell invasion (Lai Y.-J. et al., 2010), as well as TGF-β1, integrin alpha-V (ITAV), and cyclic AMP-responsive elementbinding protein 3 (CREB3). These proteins are associated with cell migration and contribute to glioblastoma’s invasive properties.

## Conclusion

A targeted metabolomic screening of glioblastoma and peritumoral tissues from cancer patients was conducted using the LC-MS/MS method. Bioinformatic analysis of the resulting metabolic profiles, employing statistical methods and gene network reconstruction, provided valuable insights into the mechanisms underlying glioblastoma development and progression. The study revealed altered metabolism of coenzyme A (CoA) and related metabolites in glioblastoma tissues, distinguishing them from peritumoral cells. Reduced levels of CoA and malonyl-CoA in glioblastoma tissues suggest increased β-oxidation of fatty acids and enhanced resistance to oxidative stress in glioblastoma cells.

Additionally, elevated ceramide levels in tumor tissue indicate potential modifications in the enzymatic activity involved in ceramide synthesis and degradation, which may be linked to tumor growth. These findings suggest that disruptions in lipid metabolism, particularly involving CoA and ceramide pathways, play a crucial role in glioblastoma pathogenesis. Such alterations highlight potential therapeutic targets for developing novel treatments aimed at the disrupted metabolic pathways in tumor cells. In particular, inhibition of key enzymes, such as serine palmitoyltransferase and sphingomyelinase, emerges as a promising strategy to reduce cell viability and potentially prevent further growth of glioblastoma cells.

Thus, the findings of this study enhance our understanding of the metabolic characteristics of glioblastoma and offer new opportunities for developing targeted therapeutic strategies focused on disrupting lipid metabolism in tumor cells. Future research on specific metabolic alterations across different glioblastoma subtypes, alongside the development and evaluation of inhibitors targeting key enzymes, could contribute significantly to advancing treatment options for this disease.

## Conflict of interest

The authors declare no conflict of interest.
